# P-410. Enhancing the Clinical Impact of mcfDNA-NGS through an ID-Physician Led Diagnostic Stewardship Approval Protocol in a Quaternary Pediatric Hospital

**DOI:** 10.1093/ofid/ofaf695.627

**Published:** 2026-01-11

**Authors:** Jordan Cox, Muayad Allali, John C Christenson, LaKeisha Boyd, Sarah Fortna, Jack G Schneider

**Affiliations:** Indiana University School of Medicine, Indianapolis, IN; Indiana University School of Medicine, Indianapolis, IN; Indiana University School of Medicine, Indianapolis, IN; Indiana University, Indianapolis, Indiana; Indiana University School of Medicine, Indianapolis, IN; Indiana University School of Medicine, Indianapolis, IN

## Abstract

**Background:**

Microbial cell-free DNA next-generation sequencing (mcfDNA-NGS) is a noninvasive diagnostic tool capable of rapidly detecting a broad range of pathogens and resistance genes. While promising, its clinical utility has shown mixed results. To optimize its use, we implemented an ID physician-led diagnostic stewardship approval protocol with mandatory ID consultation to guide test ordering, interpretation, and antimicrobial management.

**Figure:**
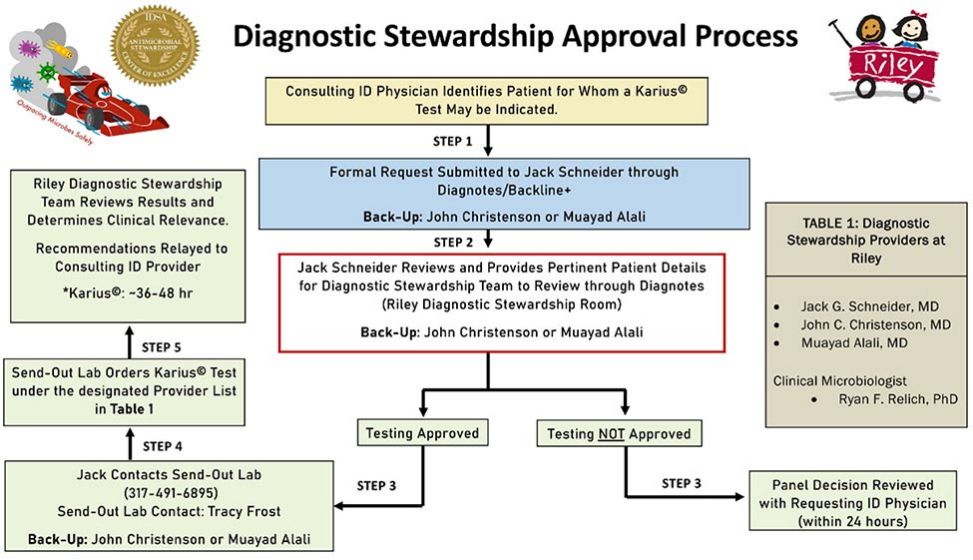
Diagnostic Stewardship Approval Protocol for Karius© Testing

**Methods:**

From February 2022 to March 2025, we conducted a prospective, single-center study at Riley Hospital for Children to assess the clinical utility of mcfDNA-NGS testing under an ID physician-led diagnostic stewardship approval protocol [Figure 1]. A panel of three infectious diseases physicians reviewed all test requests before approval, and the clinical relevance and impact of the results were subsequently evaluated.

**Results:**

A total of 54 mcfDNA-NGS tests were approved from 50 patients (median age: 9.5 years), with 64.8% yielding clinically significant results. In 24.1% of cases, mcfDNA-NGS provided the sole microbiologic diagnosis. Overall, 53.7% of tests led to clinical management changes, including earlier diagnosis (14.8%), initiation of targeted antimicrobial therapy (27.8%), and antimicrobial de-escalation (27.8%). Among patients with immunocompromising conditions (n=25), 76.0% of test results were clinically significant, and 48.0% led to clinical management changes: earlier diagnosis (20.0%), targeted therapy initiation (24.0%), and de-escalation (28.0%). Notably, the diagnosis of culture-negative endocarditis (n=9) had the highest yield, with all results leading to clinical management changes. There were no instances in which mcfDNA-NGS testing prompted unnecessary antibiotic therapy or invasive procedures. Additionally, 20.7% of test requests were deemed unnecessary and were canceled following stewardship review.

**Conclusion:**

An ID physician-led diagnostic stewardship approval protocol enhanced the clinical utility of mcfDNA-NGS testing in a pediatric cohort. These findings support broader implementation and call for multicenter studies to further refine diagnostic stewardship strategies that maximize the clinical value of mcfDNA-NGS.

**Disclosures:**

Jack G. Schneider, MD, MiraVista Diagnostics: Advisor/Consultant|Qlinea: Advisor/Consultant

